# Real-World Use of Alirocumab: Experience from a Large Healthcare Provider

**DOI:** 10.3390/jcm12031084

**Published:** 2023-01-30

**Authors:** Avishay Elis, Cheli Melzer Cohen, Gabriel Chodick

**Affiliations:** 1 Department of Internal Medicine “C”, Beilinson Hospital, Rabin Medical Center, Petah-Tikva 4941492, Israel; 2 Sackler School of Medicine, Tel Aviv University, Tel Aviv 6997801, Israel; 3 Maccabi Institute for Research and Innovation, Maccabi Healthcare Services, Tel-Aviv 6812509, Israel

**Keywords:** real-world evidence, alirocumab, anti-PCSK9 monoclonal antibodies, target levels, LDL-C

## Abstract

With the emerging use of anti-PCSK9 monoclonal antibodies for lowering low-density lipoprotein cholesterol (LDL-C) levels, real-world evidence (RWE) is needed to evaluate drug effectiveness. This study aimed to characterize new users of alirocumab and evaluate its effectiveness in achieving LDL-C target levels. Included were patients initiating treatment with alirocumab from 1 August 2016 to 1 May 2020, with blood lipids evaluations during baseline (180 days prior to therapy initiation) and after 120 (±60) days of follow-up. Patients with treatment intensification during the follow-up period were excluded. LDL-C change from baseline and reaching LDL-C target levels, according to 2019 ESC/EAS guidelines, were evaluated. Among 623 included patients, 50.2% were men, the mean age was 65 years (±9 y), 62% were classified as very-high risk, and 76% had statin intolerance. During the follow-up, 65% (n = 407) were treated only with alirocumab. In 90% the initiation dose was 75 mg, and 21% were up-titrated. Alirocumab was associated with a 31.7% reduction in LDL-C, with 20.5% of patients reaching target levels. In this RWE study, alirocumab was used primarily as a single agent for eligible patients. Suboptimal use and adherence to therapy may have led to a lower LDL-C reduction compared to previous RCTs and most reported real-world studies.

## 1. Introduction

Elevated levels of serum cholesterol, especially low-density lipoprotein cholesterol (LDL-C), correlate with the risk of coronary heart disease, stroke, peripheral vascular disease, and atherosclerosis [1]. Based on the subject’s risk, LDL-C target levels are determined and lipid-lowering therapy, mainly statins, is recommended [2]. 

Statin therapy, with or without ezetimibe, is considered to be highly effective for decreasing LDL-C levels [1]. However, in patients who do not reach the target levels on maximal tolerated statins and ezetimibe, either due to undesirable side effects or very-high LDL-C levels, human monoclonal antibodies against the proprotein convertase subtilisin/kexin type 9 (PCSK9) should be considered [3]. Other therapy options are considered less effective as shown in Table 1.

The use of anti-PCSK9 monoclonal antibodies (anti-PCSK9 mAb.) is based on randomized clinical trials (RCTs) which include specified populations. These trials evaluated the change in LDL-C levels from baseline up to 24–78 weeks, and all showed a significant reduction of approximately 60% [5,6]. Cardiovascular outcome trials (CVOT) documented a significant reduction in various events with good tolerability [7] and demonstrated a reduction in major adverse cardiovascular events in stable patients with atherosclerotic cardiovascular disease (ASCVD) and following acute coronary syndrome (ACS) [8,9,10].

Two anti-PCSK9 mAb agents, alirocumab and evolocumab, were introduced in Israel in August 2016. These medications are approved for high and very high cardiovascular (CV) risk patients, who cannot attain LDL-C target levels. The definition of high and very-high risk in this study is according to the 2019 European Society of Cardiology (ESC)/European Atherosclerosis Society (EAS) dyslipidemia guidelines [11]. In Israel, both agents are included in the national basket of covered services but require prior authorization and patients’ copayment.

As the use of these agents is emerging, there is a need for real-world data concerning their treatment patterns, effectiveness, and tolerability. A recently published meta-analysis of 67 studies, involving 28,266 patients using PCSK9 inhibitors in a clinical setting, indicated a mean reduction in LDL-C of 87 mg/dL (corresponding to 54% decrement from baseline) over a mean follow-up was 8.9 months with only 11% discontinuing therapy. This mean reduction is comparable to or greater than the mean reduction in LDL-C reported in RCTs (66.9 mg/dL and 60 mg/dL in alirocumab) [12]. The aims of this study were, therefore, to depict the demographic and clinical characteristics of patients under the treatment with alirocumab in a real-world setting [13]; to validate the reported high therapy effectiveness in terms of lowering LDL-C levels and reaching target levels as well as to document tolerability.

## 2. Material and Methods

The study utilized deidentified data from the Maccabi Healthcare Services (MHS) central computerized database. MHS is the second-largest state-mandated health provider in Israel, serving more than 2.6 million members (26% of the general population) and is a representative sample of the Israeli population. This fully computerized database captures all information on patient interaction (including demographics, diagnoses, inpatient and outpatient visits, procedures, imaging, medications prescriptions, and actual dispenses as well as laboratory data). Based on this information, MHS developed daily updated registries for major chronic diseases, such as hypertension, cardiovascular diseases (CVD), and diabetes mellitus (DM), to improve disease management and quality of care [14].

### 2.1. Study Population and Design

In this retrospective, observational single-arm study, we identified adult MHS members who have initiated treatment with alirocumab from 1 August 2016 to 1 May 2020. The date of the first alirocumab dispensing was defined as the study index date. Additional study inclusion criteria included: having at least two dispenses of alirocumab within 90 days from the index date, a blood lipids laboratory test within 180 days before the index date, and at least one additional one up to 120 days after it. Excluded were patients who were treated with anti-PCSK9 mAb other than alirocumab within 180 days prior to or after the index date as well as patients that had intensified their statins dose or initiated treatment with ezetimibe after the index date. In addition, patients with less than a year of membership in MHS were excluded to ensure a sufficient time of documented medical history in the computerized data systems.

Treatment discontinuation was defined as the first time having a gap of 90 days or more between two purchases, taking into account the treatment duration in the previous purchase. Adherence to alirocumab was assessed as the proportion of days covered (PDC) and medication possession ratio (MPR). PDC was calculated as the number of supply days during the follow-up period divided by the total number of days in the follow-up period. MPR was calculated as the total sum of days of supply divided by time on therapy.

The study protocol was granted approval by the MHS institutional review board and a waiver of informed consent.

### 2.2. Study Variables

The study population was characterized at baseline and presented by patients’ demographics, anthropometric measurements, comorbidities, blood lipid levels, and concomitant medications. Anthropometric measurements and laboratory measurements at baseline were defined as the last measurement within 180 days prior to the index date. CVD, hypertension, DM, chronic kidney disease (CKD) stage, and cancer co-morbidities were based on enrollment in MHS chronic disease registries, while revascularization procedures (coronary artery bypass graft and percutaneous coronary intervention) were based on procedures conducted during the baseline period. Other comorbidities were based on diagnoses coded by the International Classification of Diseases Version 9 with clinical modifications (ICD-9 CM) codes captured in primary and secondary care visits.

CV risk at baseline was defined as followed: Very-high risk: having one of the following: (a) included in MHS’ cardiovascular registry (includes patients with ischemic heart disease, cerebrovascular disease, and peripheral vascular disease cardio sub-registries); (b) included in diabetes mellitus registry and having diabetes complications based on ICD-9 codes (neuropathy: ICD-9 CM codes 250.6, 354.×, 355.×, 357.2; nephropathy: ICD-9 CM codes 250.4, 583.81, 585.× or estimated glomerular filtration rate (eGFR) < 60 mL/min/1.73 m^2^; retinopathy: ICD-9 CM codes 250.5, 362.×, 366.41, 365.44, 362.07); (c) having Type 1 diabetes mellitus for more than 20 years; (d) included in MHS’s CKD registry in stage 4 or worse or eGFR < 30 mL/min/1.73 m^2^; (e) diagnosed with familial hypercholesterolemia (FH: ICD-9 CM code 272.0) and included in peripheral vascular registry (PVD) registry. High CV risk: having one of the following: (a) total cholesterol > 310 mg/dL, blood pressure higher than 180/110 mmHg, or LDL-C > 190 mg/dL; (b) Diagnosed with FH but not included in PVD registry; (c) patients included in MHS’s diabetes registry for more than 10 years, but without documented diabetes complications; (d) included in MHS’s CKD registry in stage 3. Otherwise, patients were defined as lower-risk patients.

LDL-C target levels were determined according to the study definitions of CV risk based on 2019 ESC guidelines. For very-high CV risk, the LDL-C target was defined as <55 mg/dL (or non-HDL-C < 85 mg/dL when LDL-C could not be calculated). For high CV risk and lower risk, the LDL-C target was defined as <70 mg/dL (or non-HDL-C < 100 mg/dL when LDL-C could not be calculated), respectively.

Indication for alirocumab approval was defined as lack of statin effectiveness defined as not reaching LDL-C target levels after treatment with atorvastatin 80 mg or 40 mg plus ezetimibe, rosuvastatin 40 mg or 20 mg plus ezetimibe. Statin intolerance as an indication for alirocumab approval was defined as treatment with statins at doses less than those mentioned previously or not treated with statins at all prior to the study index date.

LDL-C levels evaluation for the baseline period was defined as the last measurement obtained 180 days prior to the index date and the closest measurement after 120 (±60 days) days from the index date was defined as the follow-up measurement. All LDL-C values during the study period were extracted to determine the change in LDL-C during all follow-up periods. Non-HDL-C levels were used in cases where LDL-C could not be calculated.

### 2.3. Statistical Analysis

Descriptive statistics for all baseline characteristics were presented as the mean and standard deviation (SD) for continuous variables and as counts and percentages for categorical variables. For continuous variables, statistical analyses were performed by using the Wilcoxon-signed rank test. Interaction between sub-groups was tested using the Wilcoxon sum rank test or Kruskal–Wallis test for comparisons of two categories and more than two categories, respectively. The same procedures were used to compute means and percentages in the various subgroups of patients, as defined in the protocol. Changes in LDL-C were calculated both as absolute and relative changes from baseline. When LDL-C measurement was not valid during baseline or follow-up, the change in the lipid profile was calculated using non-HDL levels.

For assessing changes in LDL-C levels over time, the following periods were defined: baseline (as described previously) and during follow-up (0–60 days, 61–120 days, 121–180 days). In each time period, patients could be included only once. If more than one measurement was recorded, the mean value was presented.

## 3. Results

**Figure 1:**
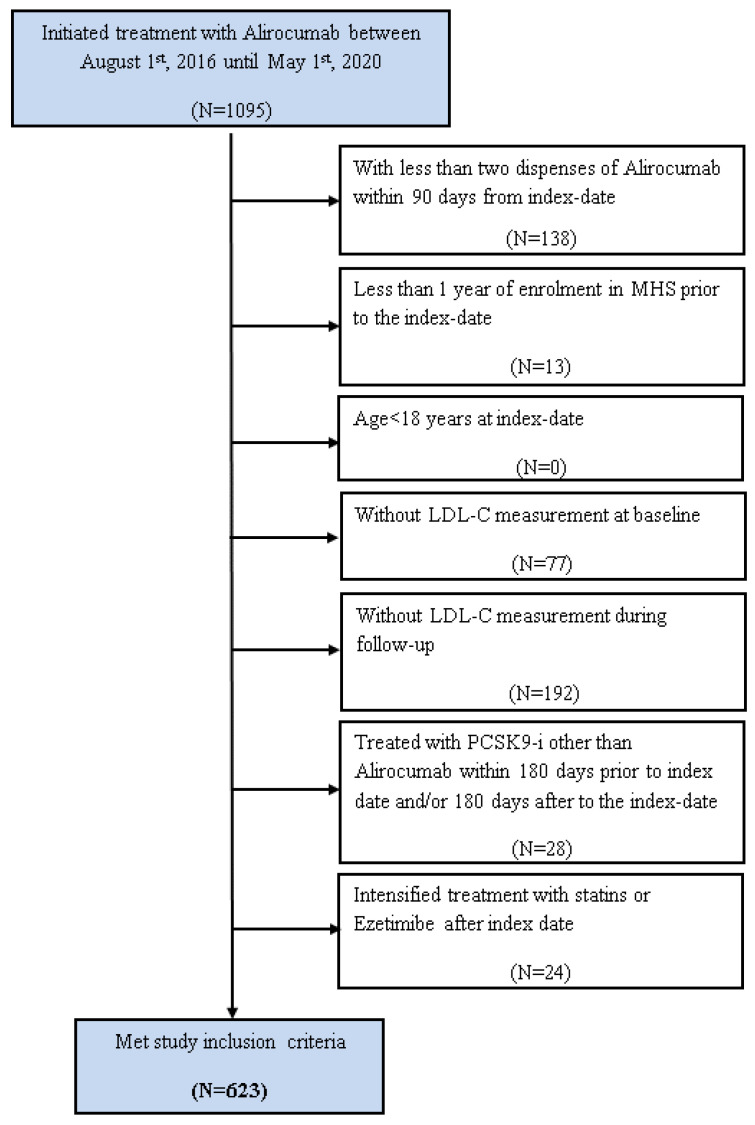
Patient flow diagram. Abbreviations: BMI: Body mass index; LDL: Low-Density Lipoprotein; MHS: Maccabi Healthcare Services; PCSK9-i: proprotein convertase subtilisin/kexin type 9 inhibitors.

A total of 1095 patients initiated treatment with alirocumab during the study period. After aligning all inclusion criteria, 623 patients were eligible (Figure 1). Patients’ characteristics are presented in Table 2. Briefly, 50.2% were men, mean age of 65 years (±9), and 62% of the study population were classified as very-high CV risk. The LDL-C mean level at the index date was 136.0 ± 48.7 mg/dL. Statin intolerance accounted for 76% of the patients initiating therapy with alirocumab. Overall, 18% (n = 115) were neither on statin nor ezetimibe at baseline. However, during the treatment follow-up, nearly half of the patients (47%) discontinued background statin and/or ezetimibe resulting in 65% (n = 407) being treated only with alirocumab as a single-lipid-lowering agent during the follow-up period.

The initiation alirocumab dose was 75 mg in 90% of the study patients. Among patients with very-high, high, and lower CV risk, the proportion of patients who initiated treatment with the dosage of 150 mg was 8.9%, 12.3%, and 9.9%, respectively. During the study period, the dose adjustment of up-titration was recorded in 129 patients (21%), and down-titration in 12 patients (1.9%). Good adherence with alirocumab, indicated by PDC and MPR of 80% or higher, was recorded in 64.4% and 69.1% of patients, respectively.

**Figure 2:**
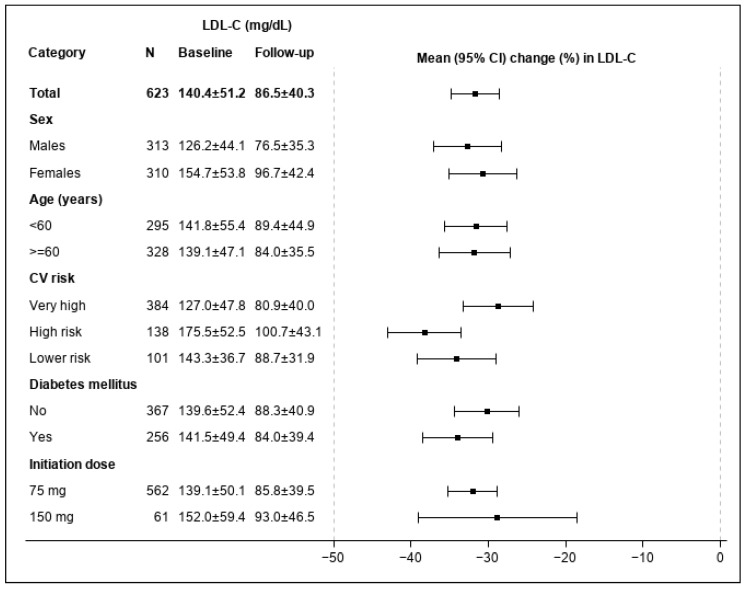
Percentage change in LDL-C within 120 (±60) days from PCSK9- i treatment initiation. If LDL-C could not be calculated during the baseline or follow-up period as a result of high triglyceride levels, then non-HDL-C was used instead. The baseline and follow up LDL-C in the graph are LDL-C (or non-HDL-C minus 30 if LDL-C could not be calculated). *p*-values for percentage change in LDL-C (or non-HDL-C when LDL-C could not be calculated) were calculated by using the Wilcoxon sign rank and were all lower than 0 (*p* < 0.001). Within group differences were tested using Wilcoxon sum rank test and the Kruskal–Wallis test for comparisons of two categories and more than two categories, respectively. All p-values between groups were higher than 0.1 in all subgroups. Abbreviations: CV: Cardiovascular; LDL-C: Low-density lipoprotein cholesterol; HDL-C: High-density lipoprotein cholesterol; PCSK9- i: anti-proprotein convertase subtilisin kexin type 9 monoclonal antibodies.

**Figure 3:**
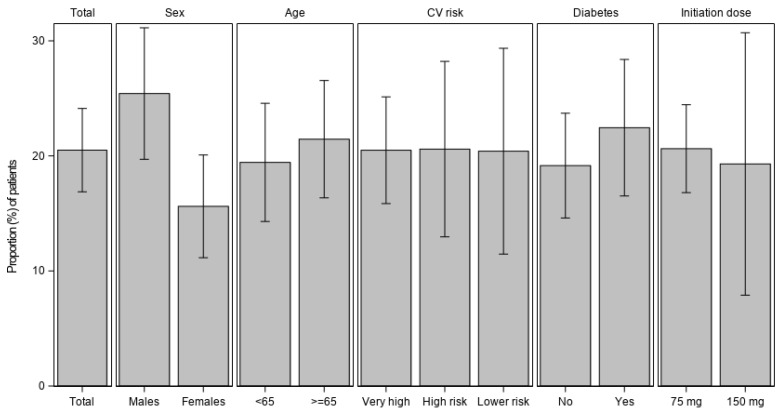
Proportion (%) of patients achieving LDL-C goals during study period. Proportion of patients and 95% confidence intervals of patients achieving LDL-C target goals according to 2019 ESC/EAS guidelines. Abbreviations: CV: Cardiovascular; LDL-C: Low-density lipoprotein cholesterol.

During the study period, alirocumab therapy was associated with a significant (*p* < 0.001) reduction in LDL-C levels (if not available, in non-HDL-C) of 31.7% from baseline (Figure 2). Sub-group analysis showed no significant difference in LDL-C levels reduction (P_interaction_ > 0.1) across both genders, age category, CVD risk level, underlying DM, or by initial alirocumab dosing. The proportions of patients achieving LDL-C goals are shown in Figure 3. Overall, 20.5% of study patients reached target levels during the study period.

**Figure 4:**
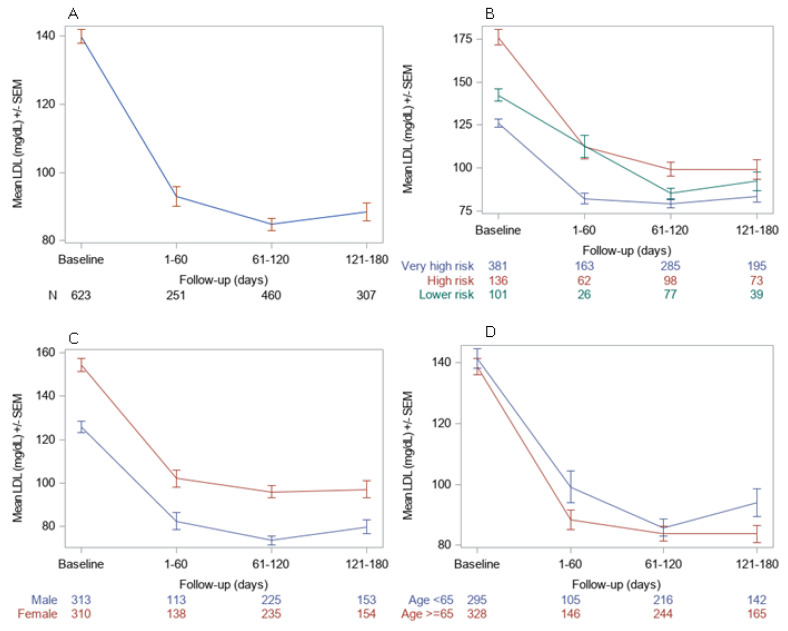
LDL-C absolute change from baseline during follow-up period by overall (**A**) and by CV risk (**B**), sex (**C**) and age (**D**) at baseline subgroups. Abbreviations: CV: Cardiovascular; LDL-C: Low-density lipoprotein cholesterol; SEM: Standard error of the mean.

Most of the reduction in LDL-C levels was achieved within 60 days of alirocumab initiation and were stable since. Similar patterns were observed across both genders, age groups, and CVD risk categories (Figure 4).

## 4. Discussion

This study aimed to characterize alirocumab treatment in the first five years after its introduction to Israel and to explore its efficacy in reducing LDL-C to target levels. It reduced LDL-C levels by approximately 32% from the baseline with most of the effect occurring within 60 days from therapy initiation. A similar LDL-C decrement was measured in both genders and across CV risk, and treatment regimens.

The indication for anti-PCSK9 mAbs use in most of our patients (76%) was statin intolerance. This finding was in accordance with other similar reports [15,16,17,18,19]. Consequently, the majority of patients (65%) in our analysis as well as in several other RWE studies (63% by Zafrir et al. [16], 70.2% by Bradley et al. [15]) were treated by anti-PCSK9 mAbs as a single-lipid-lowering agent. This is opposed to the two leading CVO studies, FOURIER, and ODYSSEY OUTCOMES [8,10] as well as most previous real-world studies. In a recently published meta-analysis of 67 RWE studies, 59% of patients on PCSK9-i had statin co-medication [12]. This may explain some of the lower effectiveness of therapy calculated in our cohort. Nonetheless, a sub-analysis of the ODYSSEY OUTCOMES study had shown that alirocumab reduces the relative risk of major adverse cardiovascular events irrespective of background statin therapy, with high efficacy among the anti-PCSK9 mAb single-agent group [20].

The level of LDL-C reduction in our study is lower than that reported with alirocumab in previous studies. In the ODYSSEY OUTCOMES [8], one year of alirocumab therapy was associated with a mean reduction of 60% [8] in LDL-C levels in the on-treatment analysis, substantially greater than in our analysis (32%) even if compared among very-high risk patients. Subsequently, while 69% of the overall population in the ODYSSEY APPRISE [21] achieved the pre-specified goal of LDL-C levels following treatment with alirocumab, only approximately 20% attained LDL-C goals in our analysis and 38% in a previous analysis from a large Israeli care provider [19] (where patients with a low adherence were excluded from the analysis which may have increased the observed therapy effectiveness). In addition to the low concurrent use of statins, differences in adherence with alirocumab may also explain the somewhat lower LDL-C reduction in our observational study compared to RCTs. For instance, while the ODYSSEY APPRISE treatment period (up to 30 months) was completed by 88.3% [21], only two-thirds of the patients in our study were covered with alirocumab for 80% or more during the study period. A similar PDC with alirocumab (64%) was reported in a claims data analysis in the US [22]. Reasons for treatment discontinuation may be due to self-injection, possible adverse events, and most probably the relatively high patients’ copayment (15% of the full price).

Moreover, only 10% of our cohort initiated treatment with 150 mg alirocumab and 21% were up-titrated during follow-up, which may explain the lower reduction in LDL-C levels in our study. Hence, persistent monitoring of therapy utilization and the patient’s lipid profile is essential to improve the quality of care together with other effective strategies that preserve adherence.

Study strengths include the use of electronic medical records of state-mandated health providers with open and free membership. A comparison of our study population characteristics with the previous study from Israel [19] shows a great similarity in the patient’s characteristics, including age and gender distribution, the prevalence of ischemic heart disease, DM, and history of statin failure in our cohort increasing the generalizability of the current cohort. Certain limitations should also be discussed. In Israel, the copayment cap for chronic medications is circa $100/month. Therefore, adherence and persistence measured in this study may not be generalizable to patients in other insurance plans where the copayment cap is higher.

The sub-analysis of the ODYSSEY OUTCOMES study had shown that alirocumab reduces the relative risk of major adverse cardiovascular events irrespective of background statin therapy, with high efficacy among the anti-PCSK9 mAb single-agent group [20].

## 5. Conclusions

In this real-world setting, alirocumab is used primarily as a single agent in an eligible Israeli population. The reduction in the use of other lipid-lowering medication during follow-up and adherence patterns may have led to lower LDL-C reduction than observed in RCTs and most reported real-world studies. Further studies are warranted to clarify these issues.

## Figures and Tables

**Table 1:** LDL-C reduction by Commonly used anti-hyperlipidemia drug [4].

Drug Class	LDL-C (% Reduction)
PCSK-9 inhibitor	38–72
HMG-CoA inhibitors	20–60
Bile acid sequestrants	15–30
Bempedoic acid	15–19
Nicotinic acid	10–25
Cholesterol absorption inhibitors	17
Fibric acid	6–20

Abbreviations: HMG-CoA: 3-hydroxy-3-methylglutaryl-coenzyme A; LDL-C: Low-density lipoprotein cholesterol; PCSK9: anti-proprotein convertase subtilisin kexin type 9. Reproduced with permission from [author], Cecil Essentials of Medicine 10th Edition; published by Elsevier, 2021.

**Table 2:** Baseline characteristics of included patients (N = 623).

Parameter	Category	n (%)
Demographics		
Males		313 (50.2)
Age (years)	<55	93 (14.9)
	55- < 65	202 (32.4)
	65- < 75	254 (40.8)
	75+	74 (11.9)
	Mean ± SD, n	64.7 ± 9.1, 623
Smoking status	Current smoker	55 (8.8)
	Past smoker	9 (1.4)
	Never Smoker	276 (44.3)
	Unknown	283 (45.4)
BMI (kg/m^2^)	Mean ± SD, n	28.9 ± 4.7, 493
CV risk (2019 ESC/EAS guidelines)	Very high	384 (61.6)
	High	138 (22.2)
	Lower risk	101 (16.2)
Co-morbidities		
High-blood pressure registry		389 (62.4)
Cardiovascular disease *		349 (56.0)
Diabetes mellitus		256 (41.1)
Pure hypercholesterolemia		92 (14.8)
Congestive heart failure		26 (4.2)
Peripheral vascular disease		55 (8.8)
Retinopathy		51 (8.2)
Peripheral neuropathy		40 (6.4)
Cancer		133 (21.3)
Cholesterol lowering therapy		
Statin		409 (65.7)
Ezetimibe		432 (69.3)
Laboratory/clinical measurements		Mean ± SD, n
Total cholesterol (mg/dL)		220.2 ± 58.1, 623
Low-Density Lipoprotein (mg/dL)		136.0 ± 48.7, 562
High-Density Lipoprotein (mg/dL)		50.0 ± 12.6, 623
non-HDL Cholesterol (mg/dL)		170.5 ± 54.6, 623
e-GFR (mL/min/1.73 m^2^)		82.4 ± 18.0, 590
Systolic blood pressure (mmHg)		129.8 ± 14.2, 608
Diastolic blood pressure (mmHg)		75.6 ± 9.1, 608

* Included in CVD major registry which includes the following: Ischemic heart disease, Peripheral vascular disease, Myocardial infarction, underwent cardiac revascularization procedure or cerebrovascular disease. Abbreviations: BMI: Body mass index; CV: Cardiovascular; HDL: High-Density Lipoprotein; e-GFR: estimated glomerular filtration rate.

## Data Availability

Data in this study is not available to share for confidentiality reasons. Queries about the data should be directed to the corresponding author.
